# Demonstration of velocity selective myocardial arterial spin labeling perfusion CMR

**DOI:** 10.1186/1532-429X-18-S1-P98

**Published:** 2016-01-27

**Authors:** Terrence R Jao, Krishna S Nayak

**Affiliations:** 1 Biomedical Engineering, University of Southern California, Los Angeles, CA USA; 2 Electrical Engineering, University of Southern California, Los Angeles, CA USA

## Background

Arterial spin labeling of the heart has been shown to estimate myocardial perfusion and perfusion reserve at a single short-axis slice for coronary artery disease assessment. However, current spatial labeling methods suffer from transit delay effects when imaging is extended to more than a single slice. Velocity selective (VS) labeling is a promising alternative that does not suffer from transit delay effects.

## Methods

**Figure 1:**
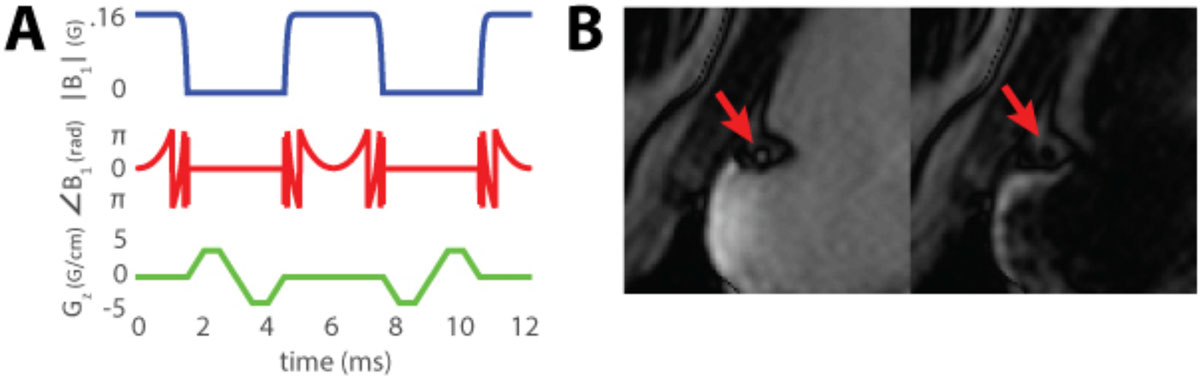
**A. the Velocity selective pulse is made using a symmetric BIR4 for reduced eddy current sensitivity and bipolar gradients to prevent spatial signal modulation in static tissue**. **B**. Coronary arterial blood (red) is saturated by VS labeling at mid-diastole with a velocity cutoff of 10 cm/s. **Left:** Gz "off" (T_2_ weighting only) **Right:** Gz "on" (T_2_ weighting + velocity selective saturation).

Eight healthy volunteers were scanned using a 3T GE Signa Excite HD scanner with an 8-channel cardiac coil. Myocardial ASL measurements were made at a single short axis slice using both VSASL and conventional flow alternating inversion recovery (FAIR) ASL as a reference. VS labeling was performed using an adiabatic BIR4 pulse with bipolar gradients as shown in Figure 1A. Labeling was achieved by saturating all spins above a cutoff velocity of 10 cm/s to target coronary blood velocity. Triple inversion recovery was used to suppress a range of myocardial T1s between 1250 ms and 1450 ms for background suppression. 6 breath-held labeled/control image pairs were acquired per subject. Myocardial blood flow (MBF), physiological noise (PN), and temporal SNR (TSNR = MBF/PN) were measured within the left ventricular myocardium ROI.

## Results

Figure 1B shows that the VS labeling pulse was successful in saturating blood within the coronary artery. MBF and PN measurements from VSASL and FAIR were 1.69 ± 0.84 ml/g/min and 2.22 ± 0.57 ml/g/min respectively in eight volunteers. This corresponds to a TSNR of 2.01 and 3.89 respectively. VSASL underestimated MBF by 23.8% when compared to FAIR, which may be due to a signal loss of 15% from inversion inefficiency in background suppression combined with a T2 signal loss of 7% from the 12 ms VS pulse. We suspect that higher PN in VSASL is from spurious labeling of myocardium, which can be further reduced by more consistent background suppression. Low TSNR will be addressed by further sequence improvements that explore different cutoff velocities and velocity labeling directions. In a single volunteer, we performed an additional FAIR experiment with a thicker inversion slab (FAIR-TS) to simulate whole heart coverage. MBF and PN measurements from VSASL, FAIR, and FAIR-TS were 1.38 ± 0.58 ml/g/min, 0.88 ± 0.37 ml/g/min, and -0.04 ± 0.24 ml/g/min respectively. FAIR-TS has a large transit delay and is unable to estimate MBF while VSASL does not suffer from transit delay effects.

## Conclusions

We have successfully labeled coronary blood based on velocity and demonstrated that VSASL is sensitive to myocardial perfusion. We believe that VSASL has the potential to become a more sensitive labeling scheme than spatial labeling sequences because of its insensitivity to transit delay and because it is inherently compatible with whole heart coverage.

**Figure 2:**
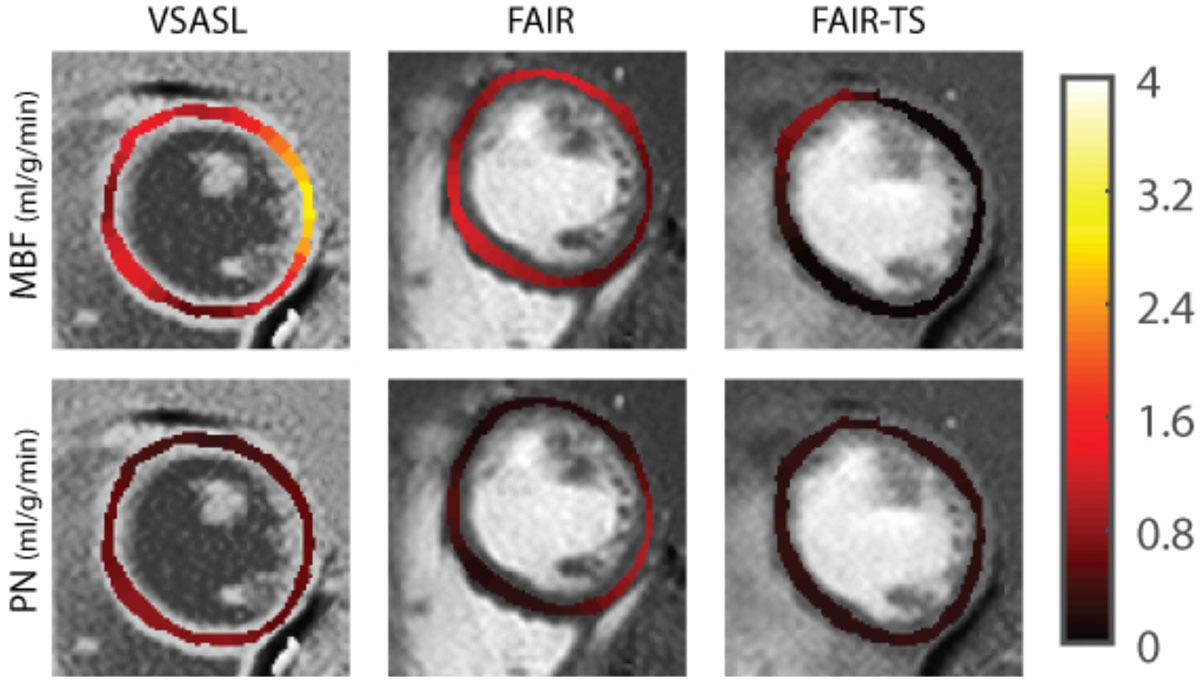
**MBF and PN measurements from VSASL, FAIR, and FAIR-TS with a thicker inversion slab to simulate whole heart coverage in a single volunteer**. Both VSASL and FAIR are able to measure MBF. The region of high MBF on the lateral wall in VSASL is possibly due to spurious myocardial labeling. While VSASL has higher PN than FAIR, it is compatible with whole heart coverage because it does not suffer from transit delay effects. FAIR-TS is unable to measure MBF because the thickened inversion slab imparts a large transit delay.

